# Personalized Genotype‐Based Approach for Treatment of Phenylketonuria

**DOI:** 10.1002/jimd.70067

**Published:** 2025-07-29

**Authors:** Polina Gundorova, Behnam Yousefi, Mathias Woidy, Malcolm Summer Rose‐Heine, Robin Khatri, Viviane Kasten, Stefan Bonn, Ania Carolina Muntau, Soeren Waldemar Gersting

**Affiliations:** ^1^ University Children's Research, University Medical Center Hamburg‐Eppendorf (UKE) Hamburg Germany; ^2^ German Center for Child and Adolescent Health (DZKJ), Partner Site Hamburg, University Medical Center Hamburg‐Eppendorf (UKE) Hamburg Germany; ^3^ Institute of Medical Systems Bioinformatics, Center for Biomedical AI (bAIome), Center for Molecular Neurobiology (ZMNH), University Medical Center Hamburg‐Eppendorf Hamburg Germany; ^4^ University Children's Hospital, University Medical Center Hamburg‐Eppendorf (UKE) Hamburg Germany

**Keywords:** misfolding, phenylalanine hydroxylase, phenylketonuria, sapropterin dihydrochloride, tetrahydrobiopterin

## Abstract

Extensive studies have examined the clinical manifestations, pathogenic mechanisms, and genetic variations of phenylketonuria (PKU) across different populations, resulting in a substantial collection of molecular genetic data on the phenylalanine hydroxylase (*PAH*) gene and its variants. However, many genotypes are associated with a range of clinical phenotypes, as well as variable responsiveness to sapropterin, presenting ongoing challenges for effective treatment. To address this, we enhanced the PAH activity landscapes method by incorporating high‐throughput techniques, including automated pipetting, integrated data processing via Gaussian modeling of 3D surfaces, and bioinformatics analyses with robust quality control. Using PAH activity landscapes, we visualized PAH enzymatic function across 99 common *PAH* genotypes under varying metabolic and therapeutic conditions. This deep functional phenotyping approach enabled us to identify distinct genotype subpopulations by using consensus clustering, correlate them with clinical phenotypes, and propose subpopulation‐specific treatment protocols. Our findings suggest that clinical phenotypes can be predicted and treatment regimens can be adjusted based on residual PAH function profiles. To further support personalized treatment strategies, we revised our publicly accessible *PAH genotype & activity landscapes database* to share the latest insights into PAH function and patient phenotypes—namely residual enzyme activity and responsiveness to sapropterin as conveyed by two alleles. This resource underscores the translational significance of functional research in PKU and offers a practical tool to support personalized treatment in clinical settings.

## Graphical abstract

Modeling the residual enzyme activity of *PAH* genotypes allows for personalized prediction of patient phenotype and BH_4_‐treatment response.

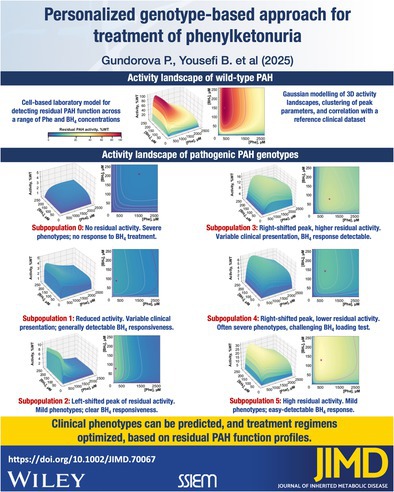

## 1 Introduction

Phenylketonuria (PKU) is a prevalent inborn error in phenylalanine (Phe) metabolism caused by a deficiency in phenylalanine hydroxylase (PAH). Despite decades of research into its clinical features, pathogenesis, genetic variability, and extensive molecular data on *PAH* variants, predicting how specific *PAH* genotypes translate into clinical outcomes and treatment responses remains challenging. To address this, we aim to study enzyme behavior in *PAH* homo‐ and compound‐heterozygous genotypes.

### 1.1 PKU Pathogenesis and Treatment

PKU results from defects in the PAH enzyme, impairing the hydroxylation of Phe to tyrosine (Tyr) and resulting in elevated blood Phe concentrations with neurotoxic effects [1]. Early initiation of a Phe‐restricted diet prevents severe symptoms, but requires strict lifelong adherence. However, long‐term compliance is difficult, risking malnutrition and reducing quality of life [2].

Biallelic *PAH* variants reduce enzyme activity. Tetrahydrobiopterin (BH_4_), a natural PAH cofactor, was first found to lower blood Phe [3] and improve PAH function, leading to the definition of the BH_4_‐responsive PAH deficiency phenotype [4]. BH_4_ acts as a chaperone, classifying PKU as a protein misfolding disorder [5]. *Sapropterin dihydrochloride*, a pharmacological BH_4_ analog, offers non‐invasive treatment for responsive patients, enabling reduced dietary restrictions and, in some cases, an unrestricted diet. This therapy improves quality of life, but is only effective if residual PAH enzyme is present. Its effectiveness depends on the genotype, and even among patients with mild *PAH* variants, the response can be inconsistent [6].

### 1.2 Functional Studies of PAH

Functional studies are essential for translating genetic knowledge into treatments for inherited diseases. Understanding enzyme behavior in mutated protein variants enables efforts to restore activity in vitro and potentially in vivo. Development of recombinant PAH activity landscapes—3D models mapping enzyme function based on substrate Phe and cofactor BH_4_ concentrations ([Phe] and [BH_4_]) [7]—revealed that PAH activity depends not only on genotype but also on metabolic state and BH_4_ dosage.

However, a knowledge gap remains regarding enzymatic function associated with *PAH* genotypes, with limited data for single variants expressed in diverse cellular systems [6, 8, 9, 10]. The functional PAH enzyme complex arises from transcripts from both alleles, comprising a heterotetramer assembled from two identical or nonidentical homodimers [11]. *PAH* patient genotypes show frequent compound heterozygosity [8], and the variability of the functional PAH enzyme complex, including enzymatic function, protein half‐life, and aggregation propensity, complicates the prediction of mutant PAH complex activity. In vivo analysis of PAH function using patient tissue samples is not feasible because PAH expression is limited to liver and kidney tissues. Therefore, a consistent model is required to evaluate the function of PAH enzyme expressed from two alleles, reflecting patient genotypes.

Bicistronic prokaryotic expression systems enable the study of PAH heterotetramers and interallelic complementation effects [12], but they are not suitable for investigating a wide range of genotypes, and their use is complicated by degradation of tagged recombinant proteins and expression difficulties for certain variants [13]. Eukaryotic systems, supported by natural chaperones, offer better stability and more accurate modeling of PAH behavior. A dual‐expression eukaryotic vector system for studying interallelic complementation [14] was further transferred to activity landscape format with reliable co‐expression of both alleles in a 1:1 ratio [15], establishing a robust model for analyzing various genotypes. This method not only enables a detailed exploration of PAH function across different [Phe] and [BH_4_], but also offers insights into genotype–phenotype correlations and their implications for personalized treatment strategies.

### 1.3 Personalized Approach for PKU Treatment

Functional studies are crucial for determining the optimal treatment strategy in patients with PKU [16]. Positive BH_4_ loading test predicts successful long‐term therapy. However, responses can vary, requiring test adjustments, such as extended duration, higher BH_4_ dosage, or specific [Phe] [17], otherwise posing risks of false‐negative results. A comprehensive approach is needed to personalize testing and dietary strategies. Recent PKU research supports individualized treatment, but applying unsystematized knowledge remains challenging. This study presents a structured public resource to support personalized care, providing a workflow to analyze and identify subpopulations of patients based on their PAH residual function profile, enabling genotype‐guided therapy.

## 2 Materials and Methods

### 2.1 Overexpression of PAH Protein

*PAH* gene variants were named according to reference sequences NC_000012.11, NM_000277.3, NP_000268.1. A variant library (Table S1) was created using site‐directed mutagenesis (QuikChange, Stratagene, USA) and cloned into a pEF‐DEST51 vector (Invitrogen, USA) containing a Kozak sequence, modified *PAH* cDNA, and a stop codon. Missense and nonsense variants reflected predicted amino acid changes. Two splice variants were cloned based on previously reported effects: p.Gln355_Tyr356insGlyLeuGln for c.1066‐11G> A [18] and p.Asn401Ter for c.1315 + 1G> A [19]. For each replicate, 4 million COS‐7 cells were electroporated (Amaxa Nucleofector II, Lonza, Switzerland) with 4 μg of DNA (2 μg per allele for compound‐heterozygotes) and cultured for 26–30 h. Following trypsinization, cells were lysed (20 mM HEPES (Sigma‐Aldrich, USA), 200 mM NaCl (Chemsolute, Germany), complete mini EDTA‐free protease inhibitor (Roche, Switzerland)) through three freeze–thaw cycles.

### 2.2 PAH Activity Assay

PAH activity was measured using modified protocols from the previous studies [7, 15] in a total assay volume of 100 μL. A reaction buffer, consisting of 22.35 mM NaHEPES (Sigma‐Aldrich, USA) (pH 7.3), 1 U/μl catalase (Sigma‐Aldrich, USA), and 10 μM ferrous ammonium sulfate (Sigma‐Aldrich, USA), was combined with 10 μL cell lysate and L‐Phenylalanine. After a 5 min pre‐incubation, the reaction was initiated by adding BH_4_ (Cayman Chemical Company, USA) solution and halted after 15 min with 2 μL of 9 N HCl (Sigma‐Aldrich, USA). Tyr production was quantified by high‐performance liquid chromatography on a Hypersil ODS‐2 column (Dionex UltiMate 3000, Thermo Fisher Scientific, USA) with a running buffer (pH 4.6) containing 1.57% ammonium hydroxide (Sigma‐Aldrich, USA) and 2% acetic acid (AppliChem, Germany) at 2 mL/min.

### 2.3 PAH Activity Landscapes

Activity landscapes were generated by assaying each PAH genotype in biological triplicates using a 96‐well format. Automated pipetting (FreedomEvo150, Tecan, Switzerland) enabled parallel processing of twenty‐four 96‐well plate layouts combined into six 384‐well plates, allowing eight genotypes to be analyzed per run. Every run incorporated a positive wild‐type PAH (WT) and a negative no‐DNA control. Variant activity was normalized to WT and background‐subtracted using no‐DNA values. Implemented assay updates allowed for reduction of batch‐to‐batch variability and improvement of the results reproducibility.

[Phe] ranged from 0 to 2500 μM across plate columns and [BH_4_] from 0 to 250 μM across rows, providing higher resolution near physiological concentrations. Protein content in lysates was measured by Bradford assay (Bio‐Rad, USA; CLARIOstar, BMG LABTECH, Germany). PAH activity was calculated as pmol/mg/min of Tyr, and median values from triplicates were used to generate each genotype's activity landscape.

### 2.4 Data Analysis and Identification of Subpopulations

**FIGURE 1:**
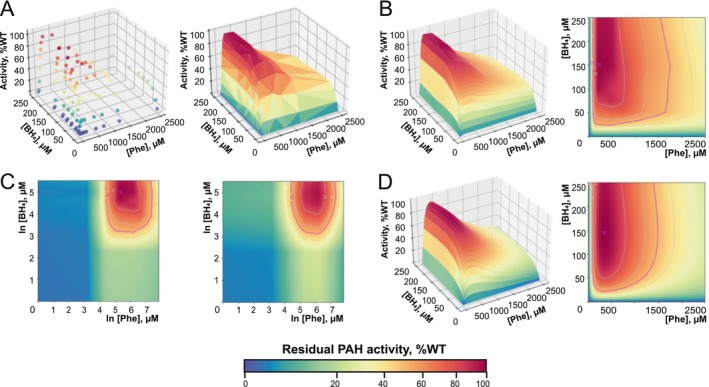
Process of data evaluation for the PAH activity landscape of human PAH WT. Data representing PAH enzyme activity at varying [Phe] and [BH_4_] were visualized using a color code (with the enzyme activity scale provided at the bottom) to depict activity as a percentage of the activity of the wild type. The sequence of graphs from left to right illustrates the data‐evaluation steps. The pink “X” on the 2D graphs indicates the peak activity position representing maximum activity. The solid pink line delineates 50% of enzyme activity, signifying its optimal working range. (A) Plotting the raw data (left) obtained from the PAH activity assay in a 96‐well format and triangular surface mesh (right) creates a non‐smoothed and non‐modeled 3D surface. (B) Data interpolation with a regular grid‐based algorithm, followed by smoothing via a Gaussian filter, allows for the creation of smoothed non‐modeled 3D (left) and 2D (right) panels. (C) The logarithmic transformation of the data creates a symmetrical surface (left) that can be fitted into a Gaussian model (right). (D) The exponential transformation of the data creates smoothed modeled 3D (left) and 2D (right) panels.

Each data sample was represented by a 12 × 8 matrix, with rows and columns corresponding to [Phe] and [BH_4_], respectively (Figure 1A). Visualization was performed using 2D/3D plots (Figure 1B) via regular grid interpolation and Gaussian filter smoothing (*SciPy* 1.12). A color code with power‐law normalization enhanced the resolution of low residual activity peaks (5%–20%).

#### 2.4.1 Gaussian Modelling

We implemented a Gaussian modeling‐based method to reduce noise and improve the quality. We fitted a 2D Gaussian function to the landscapes in log‐scaled [Phe] and [BH_4_] space (Figure 1C) and scaled it back to the original space (Figure 1D). Considering x and y as [Phe] and [BH_4_], we modeled the residual activities (*z*) as:

$$x^{\prime }=\ln\left(x\right)$$

$$y^{\prime }=\ln\left(y\right)$$

$$\hat{z}=f\left(x^{\prime },y^{\prime }\right)=a\cdot \exp\left(-0.5\;\right)\cdot \left(x-m_{x\prime }/{s_{x\prime }}^{2}\right)+\left(y^{\prime }-m_{y\prime }/{s_{y\prime }}^{2}\right)$$

where a, $m_{x\prime }$, $m_{y\prime }$, $s_{x\prime }$, and $s_{y\prime }$ are hyperparameters fitted using curve optimization (*SciPy* 1.12). From the fitted model, we extracted maximum residual activity (a), [Phe] and [BH_4_] at the peak ($\exp\left(m_{x\prime }\right)$, $\exp\left(m_{y\prime }\right)$), and then normalized the activity to the WT (%WT). Peaks occurring above 1500 μM [Phe] and lower than 3% were likely artifacts. Such landscapes were considered as having no residual activity (subpopulation 0) and were excluded from the downstream analysis.

#### 2.4.2 Quality Control

QC analysis was performed using three features: (i) the root mean squared error (RMSE) between the original and modeled landscapes, (ii) the number of peaks (NoP) in raw landscapes, and (iii) the variation between the triplicates (VT) as the maximum ratio between the range and the median value across wells. Thresholds were defined using exponential probability distribution functions (PDFs) with parameter *λ*, as the mean added to 2/*λ* (0.26, 8.4, and 0.23 for RMSE, NoP, and VT, respectively). Samples exceeding any of the thresholds were classified as low‐quality and excluded, with borderline cases reviewed manually.

#### 2.4.3 Consensus Clustering

High‐quality samples with detectable residual activity were clustered to identify genotype subpopulations. To address variability and bias in unsupervised clustering, we employed consensus clustering (R package *ConsensusClustering*) [20, 21, 22], which is robust to noise and allows estimation of optimal cluster number.

For future sample classification, we use *k*‐nearest neighbor labeling to avoid re‐clustering the entire dataset.

### 2.5 Genotype–Phenotype Correlations

The BioPKU database, a comprehensive resource for PAH genotypes and associated clinical data [6], was used to compare the in situ modeled PAH behavior with clinical phenotypes and BH_4_‐responsiveness. For each of 99 tested *PAH* genotypes, data was retrieved on:

*Clinical phenotype classification*: classical phenylketonuria [Phe] > 1200 μmol/L, mild PKU [Phe] 600–1200 μmol/L, or MHPA [Phe] < 600 μmol/L; a total of 7854 patient records.

*BH*_*4*_ *‐responsiveness*: responder, slow‐responder (combined as “responders”), non‐responder; a total of 1426 patient records.

Genotypes with > 10 patient records were included. Genotypes with variants p.Ile65Thr and p.Phe39Leu were excluded due to recent changes in the BioPKU annotations. The final dataset comprised 7756 records for phenotype correlation and 1152 for BH_4_‐responsiveness (Table S2).

## 3 Results

The *PAH* genotype dataset was created based on the selection of 42 pathogenic *PAH* variants, including all 16 of the most common variants with allele frequencies of > 1% and 26 rare variants. The selected variants accounted for 61.1% of all *PAH* pathogenic alleles [23] (Table S1). For the current study, we selected 99 *PAH* patient genotypes, of which 19 were homozygous and 80 were compound heterozygous. The summarized frequency of the 99 pathogenic *PAH* genotypes was 50.4% of all PKU patients registered in the *PAH genotype & activity landscape database* [23] (Table S2).

Building on the PAH activity landscape model [15], we further enhanced this approach by incorporating automated pipetting, integrated data processing, and bioinformatics analyses, elevating it to a high‐throughput platform. This advancement not only quadruples data output with heightened accuracy, but also conserves resources such as chemicals, consumables, and labor. Each activity landscape was produced as a result of the integration of activity assay data in a 96‐well format with gradients of [Phe] and [BH_4_] from three biological replicates (Figure 1A). Figure 1B shows 2D and 3D graphical representations of the activity landscapes based on regular grid interpolation and smoothing. To account for experimental noise and improve the quality of activity landscapes, we implemented a Gaussian modeling‐based method (Figure 1C). This approach allowed us to model the true 3D surfaces and estimate the peak parameters more precisely by considering the behavior of the entire surface (Figure 1B vs. Figure 1D). Furthermore, to ensure sample quality, we applied quality control (QC) analysis to assess the overall data quality, potential variations across experimental replicates, and the differences between observed and modeled activity landscapes.

Modeling genotype‐specific profiles of residual enzyme activity in a cellular environment allows the visualization of PAH behavior, which is usually inaccessible because its expression is limited to liver tissue. In PAH activity landscapes, enzyme activity is represented by a color code. Residual activity demonstrated an initial increase with the elevation of substrate concentrations and a subsequent reduction of activity towards higher substrate concentrations—*substrate inhibition* kinetics. The abovementioned behavior was observed for both Phe and BH_4_ and created a characteristic drop shape of a PAH activity landscape (Figure 1D). An increase in activity within the elevation of [BH_4_] in the range of 0–100 μM provides the so‐called BH_4_‐response, observed in patients with PKU. In practice, this shows how supplementing a BH_4_‐responsive patient with additional BH_4_ would increase residual PAH function, which would subsequently lower blood [Phe]. While the position of the peak of residual enzyme activity and the prominence of substrate inhibition can vary in different *PAH* genotypes, the visual presentation of BH_4_‐responsiveness in activity landscapes is defined by the presence of a residual activity peak with the activity increasing in the physiological [BH_4_] range towards the higher [BH_4_].

The PAH activity landscape for the WT demonstrated the peak of activity that was, on average, located at [Phe] = 330 ± 15 μM, [BH_4_] = 101 ± 17 μM, and the working range spanned from [Phe] 80 ± 5 μM to 1372 ± 107 μM (Figure 1D). Among the produced PAH activity landscapes for the 99 *PAH* genotypes, 18 did not show any peak of residual activity, and 81 genotypes demonstrated various residual peaks (Table S2). The presence of a peak of residual activity in the PAH activity landscape indicated a potential response to the BH_4_‐treatment. However, among patients with *PAH* genotypes demonstrating residual PAH activity, 38.9% were reported in the BioPKU database as BH_4_‐non‐responders (Table S2). This indicated that the standard BH_4_‐loading test protocol may not be efficient for different *PAH* genotypes. Identifying similarities among PAH activity landscapes for various genotypes and correlating them with clinical data allows a better understanding of the mechanism of BH_4_‐response in PAH‐deficiencies and adjustment of existing testing and treatment schemes accordingly. Therefore, we extracted the peak parameters of activity landscapes and applied clustering to extract typical patterns of PAH activity landscapes—subpopulations of PAH activity landscapes.

### 3.1 Subpopulations of PAH Activity Landscapes

**FIGURE 2:**
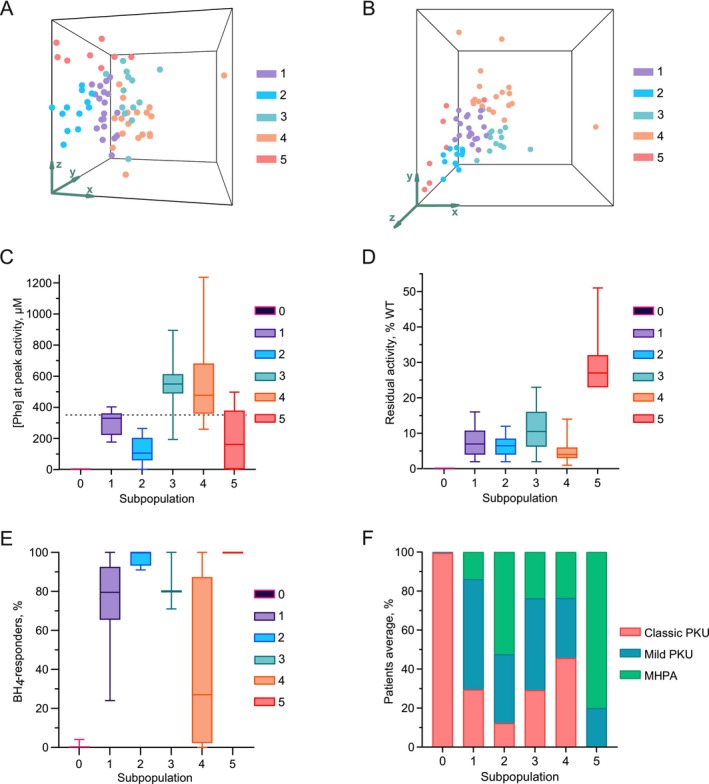
Subpopulations of PAH activity landscapes. Characteristics that define subpopulations and their clinical relevance. Box plots display the median, range from minimum to maximum, and the 25th to 75th percentiles. (A) Visual distribution of studied PAH genotypes in PAH activity landscapes based on the parameters of residual peak activity in *x*‐, *y*‐, and *z*‐side views. For better visualization, the *z*‐axis was log10 transformed. Subpopulation 0 is not shown because of the absence of a residual activity peak. Five defined subpopulations are depicted using a color code. Subpopulation 2 is located in the area of lower × (left‐shifted) and subpopulations 3 and 4 are in the area of higher × (right‐shifted). Subpopulations 3 and 4 differ in *z*‐coordinates. Subpopulation 5 groups within the highest *z*. (B) Visual distribution of studied PAH genotypes in PAH activity landscapes based on the parameters of residual peak activity *x*‐, *y*‐, and *z*‐top views. Subpopulation 0 is not shown because of the absence of a residual activity peak. Five defined subpopulations are depicted using a color code. Subpopulation 2 is located in the area of lower × (left‐shifted) and subpopulations 3 and 4 are in the area of higher × (right‐shifted). Subpopulations 3 and 4 differ by *y*‐coordinate. (C) [Phe] at peak residual activity in various subpopulations. The peak [Phe] for the PAH WT is indicated by a dashed line. Subpopulation 1 demonstrates peaks in a position similar to the WT, subpopulation 2 is characterized by left‐shifted peaks (lower [Phe]), and subpopulations 3 and 4 by right‐shifted peaks (higher [Phe]). The peaks of residual activity for genotypes in subpopulation 5 were located in the area of normal or left‐shifted peaks. (D) Maximum residual activity at the peak in various subpopulations. The two subpopulations with right‐shifted peaks, 3 and 4, differed in their maximum residual activities, with subpopulation 3 demonstrating higher values. Subpopulation 5 exhibited the highest residual peaks among all the genotypes. (E) BH_4_‐treatment response in subpopulations (clinical data sourced from BioPKU [9]). Subpopulation 0 was associated with 100% non‐responders. Genotypes from subpopulation 1 were associated with variable response rates owing to significantly reduced residual activity. In subpopulations 2 and 5, response rates close to 100% were observed owing to left‐shifted peaks and high residual peaks, respectively. Subpopulation 3 response rates were more variable owing to the right‐shifted peaks. In comparison, in subpopulation 4, right‐shifted peaks and significantly reduced residual activity led to even lower detection of BH_4_ response. (F) Clinical phenotypes in subpopulations (clinical data sourced from BioPKU [9]). Phenotypes presented as classical PKU with blood [Phe] > 1200 μmol/L, mild PKU with blood [Phe] ranging 600–1200 μmol/L, or MHPA with blood [Phe] < 600 μmol/L. Subpopulation 0 was associated with 100% classical PKU. Subpopulations 1, 3, and 4 demonstrated a mixture of possible phenotypes, with subpopulation 4 having the greatest chance of classical PKU. Subpopulations 2 and 5 represent patients with milder phenotypes, whereas subpopulation 5 does not include any patients with classical PKU.

First, we recognized the subpopulation that represented genotypes with no residual activity as subpopulation 0, which included 18 of the 99 *PAH* genotypes. These samples did not pass the QC and were not included in the cluster analysis because of the absence of a residual peak. For each of the 81 activity landscapes with a residual peak that passed the QC, we calculated: (i) the maximum residual activity normalized to the WT (%WT), (ii) the peak of activity coordinates in terms of [Phe] and [BH_4_] (μM), and (iii) the enzyme working range of 50% of the maximum activity ([Phe] μM) (Table S2). These features were used to identify and characterize subpopulations of *PAH* genotypes representing similar behaviors. Using cluster analysis, we identified five clusters (Figure 2A,B), each representing a corresponding subpopulation (1–5) of activity landscapes with various residual activity peaks. Furthermore, all subpopulations were associated with the clinical phenotype and potential BH_4_ response.

Analysis of the clustering results revealed that variation across genotypes was mainly based on two characteristics: [Phe] at peak activity (Figure 2C) and maximum residual activity (Figure 2D). According to the features defining each subpopulation, they can be characterized as follows:

0 No residual activity,
1 Reduced activity,
2 Left‐shifted peak of residual activity,
3 Right‐shifted peak, higher residual activity,
4 Right‐shifted peak, lower residual activity,
5 High residual activity.

**FIGURE 3:**
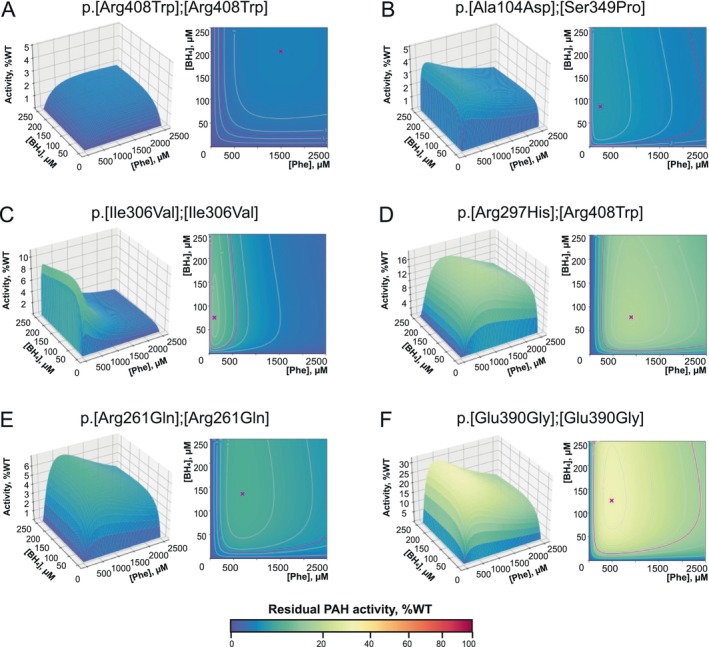
The PAH activity landscapes of genotypes corresponding to subpopulations 0–5. One example is shown for every defined subpopulation of PAH activity landscapes, and 2D and 3D panels illustrate the residual PAH activity of the corresponding genotypes normalized to the WT maximum activity. (A) Subpopulation 0: PAH activity landscapes of genotypes exhibiting no residual PAH activity; example genotype p.[Arg408Trp];[Arg408Trp]. The genotypes of this group showed no residual enzyme activity, as the designated PAH peak activity area spanning up to 1500 μM Phe displayed no discernible peaks. A heightened Tyr concentration was observed towards higher [Phe] and [BH_4_], which resulted from the natural conversion of Phe into Tyr. However, this artifact does not indicate any residual PAH activity, lies outside the metabolic space existing in human liver cells, and can only be modeled in laboratory settings. The absent peak of residual activity in subpopulation 0 directly correlates with severe clinical presentation and no response to the BH_4_‐treatment. (B) Subpopulation 1: PAH activity landscapes of genotypes with a standard peak position and reduced PAH activity, example genotype p.[Ala104Asp];[Ser349Pro]. Typically, this subpopulation displays a PAH residual activity peak that is significantly reduced in comparison with the PAH WT but remains in a similar shape and position. Some activity landscapes of this subpopulation demonstrated discernible peaks of residual activity that were not sufficiently prominent. Genotypes of subpopulation 1 are mostly associated with MHPA or mild PKU phenotypes and a positive response to the BH_4_‐treatment. (C) Subpopulation 2: PAH activity landscapes exhibiting left‐shifted peaks of residual PAH activity, example genotype p.[Ile306Val];[Ile306Val]. This subpopulation was defined by left‐shifted, narrowly peaked profiles. Notably, the working range of the enzyme predominantly falls within lower Phe concentrations. Genotypes that belong to subpopulation 2 typically lead to mHPA and a positive response to the BH_4_‐treatment. (D) Subpopulation 3: PAH activity landscapes with right‐shifted higher peaks, example genotype p.[Arg297His]; [Arg408Trp]. The PAH activity landscapes for genotypes representing this subpopulation were associated with wide right‐shifted peaks. Despite the relatively high residual peaks, a right shift often leads to undetectable residual activity below 200 μM Phe. Genotypes of subpopulation 3 could lead to more severe clinical presentations; however, the response to the BH_4_‐treatment is mostly directly detectable, due to the high residual activity peaks. (E) Subpopulation 4: PAH activity landscapes with right‐shifted lower peaks, example genotype p.[Arg261Gln];[Arg261Gln]. The PAH activity landscapes for genotypes representing this subpopulation were associated with low, wide, right‐shifted residual activity peaks. The rightward shift of both the peak and working range resulted in undetectable enzyme activity below 200 μM Phe. Genotypes grouped to subpopulation 4 are characterized by more frequent severe clinical presentation and a challenging BH_4_‐loading test. (F) Subpopulation 5: PAH activity landscapes with high residual activity, example genotype p.[Glu390Gly];[Glu390Gly]. The subpopulation includes activity landscapes of various shapes and peak locations; however, a common characteristic of this subpopulation is high residual activity. In the case of subpopulation 5, very high residual activity peaks allow for mild phenotypes and BH_4_‐response.

Examples of PAH activity landscapes for each subpopulation are shown on Figure 3; the full dataset is available on the web‐based platform at http://pah‐activitylandscapes.org/ [23].

Subpopulation 0, which showed no measurable residual activity, was excluded from the clustering analysis. Subpopulation 1 demonstrated residual activity peaks similar in position and shape to WT. In contrast, subpopulation 2 showed left‐shifted peaks, with maximum activity occurring at lower [Phe]. Subpopulations 3 and 4 were right‐shifted, with activity peaks occurring at higher [Phe] compared to WT. While subpopulation 3 had moderate residual activity, subpopulation 4 showed lower peak heights. Subpopulation 5 was characterized by high residual activity compared to other subpopulations, but with greater variability in the position of the peak along the [Phe] axis.

Clearly, different pathogenic variants of *PAH* tend to alter the enzyme activity profile in several ways. In addition to the absence of residual activity in subpopulation 0, some variants only reduced the activity to different extents, but others could also change the shape of the profiles, shifting the peak of residual activity to areas of higher or lower [Phe], as well as compressing or enlarging the working range of the enzyme. Left‐shifted peaks from subpopulation 2 (Figure 3C) were additionally characterized by a narrow working range, whereas right‐shifted peaks in subpopulations 3 and 4 (Figure 3D,E) were associated with widened working ranges. The reduction of enzyme activity at high substrate concentrations and *substrate inhibition* is known to occur in PAH [24, 25] and is observed in the majority of PAH activity landscapes. The altered position and broadness of the activity peak are provided by an enhanced or reduced substrate inhibition effect, which could be explained by changes in the surroundings and accessibility of substrate binding sites [26]. Within this hypothesis, left‐shifted narrow peaks could occur when the substrate‐binding site is more accessible, which provides for higher enzyme activity with low substrate concentrations (left‐shifted peaks), but additional binding of the substrate to the enzyme‐product complexes enhances the substrate inhibition effect (narrow activity peaks). In contrast, right‐shifted broadened peaks could occur when the substrate‐binding site is less accessible, which provides for low enzyme activity at low [Phe] (right‐shifted activity peaks), but lower binding of the substrate to the enzyme‐product complex reduces the substrate inhibition effect (broad activity peaks). Further investigation of the mechanisms by which particular pathogenic variants enhance or reduce the substrate inhibition effect would require crystal structure studies on those mutants, which, however, could be complicated by the absence of data on the Phe‐activated PAH structure [27]. Other possible strategies would involve modeling of the variant protein 3D structures *in silico*.

To investigate the clinical relevance of various changes in the shapes of activity landscapes, we compared the identified subpopulation with the data on phenotype and registered BH_4_‐response from the BioPKU database (Figure 2E,F). The reference clinical cohort resulted in 7756 patients for phenotype correlation and 1152 patients for BH_4_‐treatment correlation (Table S2). Based on the characteristics of the corresponding subpopulation, we can determine the prognosis of the patient's phenotype and BH_4_‐treatment for genotypes lacking sufficient clinical evidence in the BioPKU database. Consulting PAH activity landscapes to provide patients with blood Phe levels to match the PAH working range for particular genotypes is beneficial for optimizing BH_4_‐testing and treatment protocols.

#### 3.1.1 Genotypes With No Residual Activity: Subpopulation 0

This subpopulation, represented by 18 genotypes with homozygous or compound‐heterozygous variants, consistently exhibited no residual PAH activity (Figure 2D, Figure 3A). Notably, all possible pairwise combinations—both homozygous and compound heterozygous—of the variants p.Arg111Ter, p.Arg158Gln, p.Arg243Ter, p.Arg252Trp, p.Arg261Ter, p.Glu280Lys, p.Pro281Leu, p.Ser349Pro, c.1066‐11G>A (p.Gln355_Tyr356insGlyLeuGln, IVS10‐11G>A), p.Arg408Trp, and c.1315+1G>A (p.Asn401Ter, IVS12+1G>A) were predicted to have no residual PAH activity. Consequently, they showed no response to BH_4_ in all tested patients (Figure 2E) and were clinically represented as classical PKU (Figure 2F).

#### 3.1.2 Activity Landscapes With Reduced Residual Activity: Subpopulation 1

The largest subpopulation contained 24 genotypes that displayed a peak with location and working ranges resembling those of the WT PAH (Figure 2C, Figure 3B). However, in all these instances, activity is markedly diminished in comparison with WT, generally falling within the interval of 4%–11%. The peak of residual activity was located in the range of 220–360 μM Phe with a median of 330 μM Phe, which corresponds to a WT peak with [Phe] = 352 μM. Subpopulation 1 is formed mainly by genotypes comprising mild variants, such as p.Leu48Ser, p.Ala104Asp, p.Ala300Ser, p.Val388Met, or p.Tyr414Cys [28, 29, 30], which provide low residual PAH activity. Clinical data from the BioPKU database reflect the presence of residual activity, as 80% of patients are listed as BH_4_‐responders or slow‐responders. However, in this subpopulation, BH_4_‐response varies from 24% to 100%, and this inconsistency indicates difficulties in detecting the BH_4_‐response for some of the genotypes. Possible reasons for the inconsistent response rates could be a low residual activity provided by a certain variant, for example, p.Leu48Ser with 8% in the homozygous genotype. A similar behavior was observed in compound heterozygous genotypes that included null variants or severe missense variants, such as p.Arg408Trp, and a variant with residual activity. As p.Arg408Trp tends to promote higher aggregation [31] it induces a negative interallelic complementation effect, further reducing the already impaired residual activity provided by the second variant. For patients in this group, an extended test, such as a 4‐week test, or an increased BH_4_ dosage might be considered to detect the medication response in case of the first negative BH_4_‐loading test.

#### 3.1.3 Left‐Shifted Peaks of Residual PAH Activity: Subpopulation 2

A distinguished subpopulation consisting of 14 genotypes represented specific shifts towards lower [Phe] profiles of PAH activity landscapes (Figure 2C, Figure 3C) with a median of [Phe] at the peak of 105 μM. Additionally, the enzyme working ranges for these variants are typically confined to a narrow area, whereas the residual activity is in a relatively low range of 4%–9%. Such a profile is commonly observed in genotypes with variants p.Ala403Val and p.Ile306Val. Despite low residual activity, narrow left‐shifted profiles were associated with a 100% BH_4_‐response and mild clinical presentation (35% mild PKU and 52% MHPA) (Figure 2E,F). A possible mechanism of mild phenotypes within subpopulation 2 would be that the enzyme is active in the low range of [Phe] and effectively recycles the substrate, which does not allow for [Phe] to elevate.

Additionally, the peak of activity resided within a relatively low [BH_4_] of 55–72 μM (Figure S1). First, physiological [BH_4_] is beneficial for patients, allowing for residual PAH activity and providing mild phenotypes. Second, this peak positioning accounts for the high rates of BH_4_‐responders, suggesting that the standard BH_4_‐loading test procedure using a 20 mg/kg dosage would effectively benefit patients without yielding false‐negative results.

#### 3.1.4 Right‐Shifted Higher Peaks of Residual PAH Activity: Subpopulation 3

A subpopulation of 16 genotypes displayed PAH activity landscapes, with the peak shifted to higher [Phe] (median [Phe] = 547 μM) and a broad working range (Figures 2C and 3D). The presence of right‐shifted peaks is typically indicative of severe phenotypes and challenges in detecting BH_4_‐response. However, in subpopulation 3, this effect was partially mitigated by the higher residual activity of 6%–16% WT, which was greater than that in subpopulations 1 and 2 (Figure 2D). Despite the residual activity being higher than that in subpopulation 2, the phenotypes and BH_4_–response reflect a more severe clinical presentation. Phenotype distribution was heterogeneous and similar to that in subpopulation 1, with 29% classical PKU, 35% mild PKU, and 53% MHPA. An 80% of patients are reported as BH_4_‐responders, which reflects the possibility of failing the BH_4_‐loading test even with a high PAH residual activity. Right‐shifted peaks could lead to an insufficient BH_4_‐response while being tested with low blood [Phe], for example, when the patient is already receiving dietary treatment.

Certain genotypes, such as compounds with p.Glu390Gly and p.Asp415Asn, display relatively high residual activity and are therefore observed in milder phenotypes and linked to a potential response to the BH_4_‐treatment. Several genotypes in this subpopulation were compound heterozygous for the variant p.Ile65Thr, which has a history of contradictory reports in the literature and in the BioPKU database (earlier patients reported as slow‐responders, but later changed to non‐responders), and therefore were excluded from the analysis as unreliable data. However, PAH activity landscapes of homozygous and compound heterozygous genotypes with p.Ile65Thr demonstrated prominent right‐shifted peaks of residual activity, indicating a possible BH_4_ response. These patients, as the others with genotypes from subpopulation 3, could benefit from BH_4_ administration when having a higher Phe load to reach the residual activity that arises with higher [Phe].

#### 3.1.5 Right‐Shifted Lower Peaks of Residual PAH Activity: Subpopulation 4

This subpopulation of 19 genotypes displayed a typical picture of the PAH activity landscape when the peak was shifted towards a higher [Phe] (median [Phe] = 476 μM), broadened working range, and low residual activity from 3% to 6% (Figures 2C,D and 3E). PAH enzyme activity was not evident in the low Phe concentration range. Furthermore, these peaks often lie within higher BH_4_ concentrations (Figure S1). Mentioned characteristics are reflected by the largest share of classical PKU of 29% and the lowest BH_4_‐response rate of 27% among all subpopulations of potential BH_4_‐responders. Severe phenotypes are often reported; since the enzyme is not active at low [Phe], it cannot maintain low Phe levels. The high share of reported BH_4_‐non‐responders suggests that the standard BH_4_‐loading test procedure might not be appropriate for patients with these genotypes. It is likely that tests are often conducted when patients are already on a Phe‐restricted diet, which results in lower blood Phe levels. Consequently, the residual activity did not increase when supplemented with pharmacological concentrations of BH_4_, producing numerous false‐negative results in the BH_4_‐loading test. For such patients, it is advisable to start the BH_4_‐loading test at higher Phe levels to detect residual PAH activity. This recommendation can be integrated into the BH_4_‐treatment process. Since the peaks of activity tend to be located within the higher [BH_4_], increasing the dosage of BH_4_ might be beneficial for pikely that tests are often conducted when patients are already on a Phe‐restricted diet, which results in lower blood Phe levels. Consequently, the residual activity did not increase when supplemented with pharmacological concentrations of BH_4_, producing numerous false‐negative results in the BH_4_‐loading test. For such patients, it is advisable to start the BH_4_‐loading test at higher Phe levels to detect residual PAH activity. This recommendation can be integrated into the BH_4_‐treatment process. Since the peaks of activity tend to be located within the higher [BH_4_], increasing the dosage of BH_4_ might be beneficial for patients with genotypes of subpopulation 4.

The typical variant found in the genotypes of subpopulation 4 is p.Arg261Gln, a common pathogenic variant with an allele frequency of 9.3%, which is known to be challenging in terms of BH_4_‐therapy. Homozygous and compound‐heterozygous genotypes with p.Arg261Gln are often reported as classical PKU, and the BH_4_‐loading test does not always detect treatment response. To avoid false‐negative BH_4_‐loading test results, it is advisable to increase the Phe load at the beginning of the test and to use a higher dosage of the medication.

#### 3.1.6 Genotypes With High Residual PAH Activity: Subpopulation 5

Subpopulation 5 includes PAH activity landscapes of 8 genotypes that are heterogeneous by the shape and position of the peak, but all have high residual PAH activity ranging from 23% to 32% (Figures 2D and Figure 3F). This group includes landscapes with left‐, right‐, and non‐shifted peaks, but when reaching a certain residual activity, they all seem to provide conditions for mild phenotypes (20% mild PKU, 80% MHPA) and are in 100% of cases associated with BH_4_‐response. High residual activity guarantees a response to BH_4_ therapy or provides a mild phenotype of MHPA that does not require treatment. Notably, further research is necessary to predict whether a patient with a mild genotype will require treatment; therefore, monitoring Phe levels is recommended according to treatment guidelines.

Variants providing exceptionally high residual activity were often p.Ala403Val, p.Glu390Gly, and p.Asp415Asn. Homozygous and some compound‐heterozygous genotypes with the mentioned variants contribute to the subpopulation 5.

### 3.2 *PAH* Genotype & Activity Landscapes Database

One of the objectives of the study was to enhance and structure the *PAH genotype & activity landscape database* [23], resulting in a dedicated web application for analyzing the global distribution of *PAH* genotypes and accessing enzymatic functional data. With over 11 450 genotypes included, the database is regularly being updated with newly published population and in‐house experimental data. Researchers and clinicians can also submit their unpublished genotype data from PKU patient cohorts directly to the platform.

This application offers tools for visualizing and analyzing sequence variant distributions, exploring patient genotypes, and identifying targets for functional studies. The database has been comprehensively reorganized to include search and analysis features and integrate new functional results. It supports both research and clinical use, enabling the interpretation of *PAH* variants and access to functional data. Clinicians can directly access laboratory‐derived PAH activity landscapes, gaining insights from activity features. Each PAH activity landscape now includes a standardized verbal interpretation based on subpopulation analysis, providing clinicians with genotype‐specific guidance. Additionally, the platform fosters collaboration between researchers and healthcare providers, with the goal of improving care for patients with PAH deficiency. The initial collection of 99 PAH activity landscapes will be expanded to include frequent population‐specific genotypes and those requested by clinicians via the online interface.

## 4 Discussion

Genotype–phenotype correlations and BH_4_‐responsiveness reports in the literature and the BioPKU database are often inconsistent, making it challenging to achieve robust and predictable treatment outcomes for patients carrying the same *PAH* genotype [6, 29, 30, 32, 33, 34]. The primary objective of this study was to provide a translational personalized approach to treatment, aiming to reduce false‐negative BH_4_‐test outcomes and support the implementation of more effective BH_4_‐therapy strategies.

### 4.1 Strategies for Clinical Application of PAH Activity Landscapes

By applying a clustering algorithm to the PAH activity landscapes with measurable residual activity, we identified patterns that offer new insights into the phenotypic variability observed in PKU. These findings have potential utility in refining diagnostic workflows and individualizing treatment strategies. While PKU care is generally guided by international and national guidelines, clinical practice often varies—particularly regarding the timing of genetic diagnostics. Below, we outline two practical scenarios in which BH_4_‐treatment strategies can benefit from integrating PAH activity landscapes.

#### 4.1.1 Genetic Results Not Available at Time of BH_4_ ‐Testing

In many healthcare settings, newborns begin treatment before their *PAH* genotype is known. Since genetic testing can take time, clinicians often use this initial treatment phase to assess BH_4_‐responsiveness. By the time genetic results are received, dietary and BH_4_‐treatments may already be in place. Given the inconsistencies in BH_4_‐responsiveness reported in the BioPKU database, it is beneficial to reassess potential false‐negative BH_4_‐test results using functional data.

When initial BH_4_‐loading tests indicate non‐responsiveness, PAH activity landscapes can provide an additional layer of decision‐making support:

##### 4.1.1.1 Confirming Non‐Responsiveness

If the genotype falls into subpopulation 0 (no residual activity), BH_4_‐treatments are unlikely to be beneficial, and alternative therapies should be considered.

##### 4.1.1.2 Reevaluating BH_4_ Test Conditions

If the genotype belongs to subpopulations 1–5, a positive BH_4_‐response may be achievable under modified testing conditions. Depending on the location of the activity peak within the landscape, adjusting the patient's pre‐test Phe levels (within clinically safe limits), extending test duration, or combining both may improve responsiveness detection. Additionally, using the full approved BH_4_ dose of 20 mg/kg/day—especially in cases where testing is typically initiated at lower doses—can be beneficial. These modifications may increase the likelihood of identifying partial responders.

These recommendations are not limited to neonates or newly diagnosed patients. Older patients who were classified as non‐responders based on earlier BH_4_‐loading tests may also benefit from retrospective genotype‐based evaluation. If their genotype falls into subpopulations 1–5, a re‐test under optimized conditions may be warranted to ensure that treatment decisions reflect the most accurate functional data available.

In short, discrepancies between the BH_4_‐test results and genotype‐specific functional data should prompt a careful reexamination of test conditions.

#### 4.1.2 Genotype Available Before BH_4_ ‐Testing

An alternative scenario arises when BH_4_ becomes newly available in a clinical center, prompting loading tests in a broader patient cohort. In this case, the availability of PAH genotype data prior to testing allows for more strategic patient selection and testing design:

##### 4.1.2.1 Exclusion of Non‐Responsive Genotypes

Patients with genotypes previously identified as non‐responsive (subpopulation 0) can be excluded from testing, reducing unnecessary procedures.

##### 4.1.2.2 Adjustment of Pre‐Test Phe Levels

For genotypes with right‐shifted activity peaks (subpopulations 3 and 4), higher initial blood Phe may be necessary to detect a BH_4_‐response during testing.

##### 4.1.2.3 Test Adaptation for Low‐Activity Genotypes

Patients with significantly reduced PAH activity (subpopulations 1 and 4) may benefit from extended loading tests and/or higher doses if standard protocols do not yield positive responses.

As PKU treatment becomes increasingly individualized—particularly in the optimization of combined dietary and pharmacological therapy—PAH activity landscapes offer a valuable visualization tools. These landscapes can help clinicians, dietitians, and patients better understand genotype‐specific PAH functionality, ultimately supporting more effective personalized treatment strategies.

### 4.2 Limitations of the Study and Further Research Perspectives

Due to the high incidence of PKU and widespread newborn screening, a massive number of *PAH* variants and genotypes have been identified. While this study analyzed 99 genotypes—covering over a half of reported PKU cases—it represents only a fraction of the 2302 known *PAH* genotypes [23]. Functional testing of all variants and genotypes is impractical due to resource and cost limitations. However, the aim here was to explore correlations between in situ enzyme activity and clinical outcomes.

Further expansion of the PAH activity landscape dataset is planned, focusing on the most frequent genotypes across populations through scientific collaborations. Additionally, personalized activity landscapes could be created upon request to support clinical decision‐making.

However, the long‐term value lies in translating these experimental datasets into predictive *in silico* models. This study lays the groundwork for such tools.

The activity landscapes approach may also be applied to other enzymes involved in inherited metabolic disorders. Visualizing residual enzyme activity and detecting patterns in enzyme behavior can deepen understanding of disease mechanisms and aid in personalizing treatment strategies. With continued data accumulation, this method could enhance both therapeutic development and computational modeling.

## 5 Conclusions

PKU has been studied extensively for over nine decades, resulting in a detailed understanding of its molecular and clinical aspects. This wealth of data calls for effective tools to analyze and apply findings for patient benefit. While updating guidelines and implementing new strategies may take time, timely access to the latest research is essential to improve patient care and quality of life. A translational approach—bridging research and clinical practice—can be advanced through web‐based platforms that publish functional studies in real time, enabling treatment adjustments. Genetic diagnostics, increasingly implemented in PKU management, should not only confirm the diagnosis but also guide personalized therapy. This approach can serve as a model for individualized care in other rare diseases.

## Author Contributions

**Polina Gundorova:** writing ‑ original draft, conceptualization, data curation, funding acquisition, investigation, methodology, project administration, validation, visualization. **Behnam Yousefi:** writing ‑ original draft, data curation, methodology, software, validation, visualization. **Mathias Woidy:** writing ‑ review and editing, methodology, software. **Malcolm Summer Rose‐Heine:** investigation. **Robin Khatri:** software. **Viviane Kasten:** investigation. **Stefan Bonn:** supervision, funding acquisition, writing ‑ review and editing. **Ania Carolina Muntau:** supervision. **Soeren Waldemar Gersting:** resources, supervision, funding acquisition, writing ‑ review and editing.

## Conflicts of Interest

All authors have read the journal's policy on disclosure of potential conflicts of interest. A.C.M. and S.W.G. are shareholders of Inuiva GmbH. A.C.M. has received consulting and speaker fees from APR, BioMarin, and PTC Therapeutics. The authors declare no conflicts of interest.

## Supporting information


**Data S1:** Supporting Information.

**Figure S1:** [BH_4_] at peak residual activity across subpopulations.

**Table S1:** jimd70067‐sup‐0003‐TableS1.xlsx. *PAH* gene variants.

**Table S2:** jimd70067‐sup‐0004‐TableS2.xlsx. *PAH* gene genotypes.

## Data Availability

The data that support the findings of this study are openly available in PAH genotypes & activity landscapes database at http://pah‐activitylandscapes.org/. The data evaluation pipeline is available at the GitHub repository https://github.com/imsb‐uke/Phenylketonuria.
