# Supplementary material for: Personalized Genotype‐Based Approach for Treatment of Phenylketonuria
Source: J Inherit Metab Dis. 2025 Jul 29;48(5):e70067. doi: 10.1002/jimd.70067 (PMC12307255; doi:10.1002/jimd.70067)
Supplement: Supplementary file 1 — Data S1: Supporting Information. [file JIMD-48-0-s001.docx]

Supplementary figure 1.

[BH_4_] at peak residual activity across subpopulations.

The peak [BH_4_] for WT PAH is indicated with a dashed line. Subpopulation 2 is characterized by down-shifted peaks (lower [BH_4_]), suggesting a more profound response to BH_4_-treatment. Subpopulation 4 is characterized by up-shifted peaks (higher [BH_4_]), indicating more challenging detection of BH_4_ responsiveness and potentially requiring a higher sapropterin dosage.

Supplementary Table 1.

*PAH* gene variants.

*PAH* gene variants involved in the study, listed using standard nomenclature along with their variant allele frequencies.

Supplementary Table 1.

*PAH* gene genotypes.

*PAH* gene genotypes analyzed in the study, along with their frequencies, classification into PAH activity landscape subpopulations, peak parameters, quality control metrics, and available clinical data (phenotypic severity and BH_4_ treatment response).
