# Supplementary figures and images for: Personalized Genotype‐Based Approach for Treatment of Phenylketonuria
Source: J Inherit Metab Dis. 2025 Jul 29;48(5):e70067. doi: 10.1002/jimd.70067 (PMC12307255; doi:10.1002/jimd.70067)

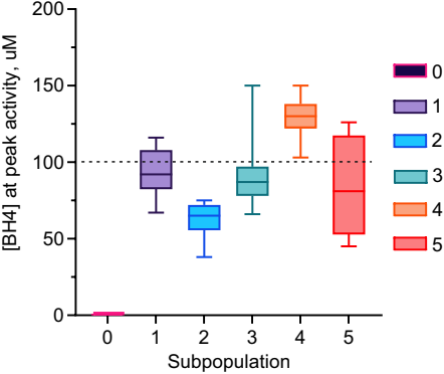

Supplement: Supplementary file 2 — Figure S1: [BH4] at peak residual activity across subpopulations. [file JIMD-48-0-s004.pdf]
